# Association of Alpha-Crystallin with Human Cortical and Nuclear Lens Lipid Membrane Increases with the Grade of Cortical and Nuclear Cataract

**DOI:** 10.3390/ijms25031936

**Published:** 2024-02-05

**Authors:** Preston Hazen, Geraline Trossi-Torres, Raju Timsina, Nawal K. Khadka, Laxman Mainali

**Affiliations:** 1 Biomolecular Sciences Graduate Programs, Boise State University, Boise, ID 83725, USA; prestonhazen@u.boisestate.edu (P.H.); geralinetrossito@u.boisestate.edu (G.T.-T.); 2 Department of Physics, Boise State University, Boise, ID 83725, USA; timsinaraju47@gmail.com (R.T.); nawalkhadka@boisestate.edu (N.K.K.)

**Keywords:** α-crystallin, single lens, cortical membrane (CM), nuclear membrane (NM), percentage of membrane surface occupied (MSO), mobility parameter, maximum splitting, hydrophobicity, cataracts

## Abstract

Eye lens α-crystallin has been shown to become increasingly membrane-bound with age and cataract formation; however, to our knowledge, no studies have investigated the membrane interactions of α-crystallin throughout the development of cataracts in separated cortical membrane (CM) and nuclear membrane (NM) from single human lenses. In this study, four pairs of human lenses from age-matched male and female donors and one pair of male lenses ranging in age from 64 to 73 years old (yo) were obtained to investigate the interactions of α-crystallin with the NM and CM throughout the progression of cortical cataract (CC) and nuclear cataract (NC) using the electron paramagnetic resonance spin-labeling method. Donor health history information (diabetes, smoker, hypertension, radiation treatment), sex, and race were included in the data analysis. The right eye lenses CM and NM investigated were 64 yo male (CC: 0), 68 yo male (CC: 3, NC: 2), 73 yo male (CC: 1, NC: 2), 68 yo female (CC: 3, NC: 2), and 73 yo female (CC: 1, NC: 3). Similarly, left eye lenses CM and NM investigated were 64 yo male (CC: 0), 68 yo male (CC: 3, NC: 2), 73 yo male (CC: 2, NC: 3), 68 yo female (CC: 3, NC: 2), and 73 yo female (CC: 1, NC: 3). Analysis of α-crystallin binding to male and female eye lens CM and NM revealed that the percentage of membrane surface occupied (MSO) by α-crystallin increases with increasing grade of CC and NC. The binding of α-crystallin resulted in decreased mobility, increased order, and increased hydrophobicity on the membrane surface in male and female eye lens CM and NM. CM mobility decreased with an increase in cataracts for both males and females, whereas the male lens NM mobility showed no significant change, while female lens NM showed increased mobility with an increase in cataract grade. Our data shows that a 68 yo female donor (long-term smoker, pre-diabetic, and hypertension; grade 3 CC) showed the largest MSO by α-crystallin in CM from both the left and right lens and had the most pronounced mobility changes relative to all other analyzed samples. The variation in cholesterol (Chol) content, size and amount of cholesterol bilayer domains (CBDs), and lipid composition in the CM and NM with age and cataract might result in a variation of membrane surface mobility, membrane surface hydrophobicity, and the interactions of α-crystallin at the surface of each CM and NM. These findings provide insight into the effect of decreased Chol content and the reduced size and amount of CBDs in the cataractous CM and NM with an increased binding of α-crystallin with increased CC and NC grade, which suggests that Chol and CBDs might be a key component in maintaining lens transparency.

## 1. Introduction

α-Crystallin is a small heat shock protein [1,2], made from two subunits (αA and αB), that comprises ~40% of the soluble lens proteins and primarily functions as a molecular chaperone to maintain long-term lens transparency by preventing misfolded protein aggregation [1,3,4,5]. However, with age and cataract formation, α-crystallin has been shown to associate with the lens membrane, leading to its insolubilization and a buildup of insoluble high molecular weight (HMW) protein aggregates within the eye lens [6,7,8]. This formation of HMW protein further leads to the scattering of light entering the eye, ultimately impairing vision and making cataracts the primary source of worldwide blindness today [9]. With age, α-crystallin can undergo various mutations [7,8,10,11] and post-translational modifications, refs. [2,12,13,14,15] reducing its chaperone-like activity [16,17,18,19,20,21,22] and the availability of free lens α-crystallin [19,23,24]. With this loss of free α-crystallin in the lens cytoplasm with age and cataract formation, there is a consequential increase in the grade of water-insoluble and HMW protein aggregates in the lens [19,23,24,25,26]. Relatedly, previous studies have found a decrease in cytoplasmic α-crystallin with the increase in membrane binding seen in aging and cataractous lenses [27,28,29], and nearly all insoluble crystallins have been shown to bind with the eye lens membrane [30]. Due to this association of insoluble crystallin with the eye lens lipid membrane in aging and cataract development, this protein–lipid interaction is believed to be a possible key mechanism of crystallin insolubilization [29,30,31]. Furthermore, α-crystallin binding to the lens membrane is thought to physically block membrane pores, creating a barrier for the diffusion of molecules such as antioxidants, in turn altering the lens environment and disrupting lens homeostasis, leading to the development of cataracts [29,32,33].

In addition to crystallin binding, it has been shown the lipid (phospholipid (PL) and sphingolipid (SL)) and cholesterol (Chol) composition changes with species [34,35,36,37], age [38,39], and cataract development [33,34,35,40,41]. In humans with age and cataracts, the lens has been shown to primarily decrease in phosphatidylcholine (PC) content while increasing in SL content [36,42], with the cortical membrane (CM) showing an increase in SL content while the nuclear membrane (NM) shows slight decreases in SL content with the development of cataracts [43]. Moreover, the Chol content within the human lens has been shown to increase with aging but conversely decreases with the development of cataracts [38,39,43]. This decrease in Chol seen with the development of cataracts is strongly associated with lens oxidation, which has been shown to truncate the lens membrane lipids, consequentially forcing Chol out of the membrane [35,44,45,46,47,48]. The human eye lens CM and NM also differs in their lipid and Chol composition, with the NM having a higher Chol and SL content but a lower PC content than the CM [36,41]. However, with the lens changes found in development of cataracts the CM develops a higher SL composition than the NM [43]. Within non-cataractous lenses of 0–20-year-olds, the NM shows a Chol/lipid molar ratio of 0.71, while the CM is lower with a Chol/lipid ratio of 0.63 [39]. However, with age-related changes, the transparent lenses of 61–70-year-olds increase in their Chol content, with the NM increasing to a Chol/lipid molar ratio of 4.4, while the CM is significantly lower with a Chol/lipid molar ratio of 1.8. This high Chol content leads to the formation of cholesterol bilayer domains (CBDs) in the lens CM and NM [39,43,49]. However, with the development of cataracts in 61–70-year-olds, the Chol/lipid molar ratio drops to 1.45 in the NM and 1.14 in the CM [43], making the decrease in Chol in the NM significantly larger than that found in the CM [38,39,43]. Due to this loss of Chol with cataract development, the amount and size of CBDs are smaller in cataractous lens CM and NM compared to the CM and NM of clear lenses [43]. Relatedly, prior in vivo studies have found that the knockout of Chol production in the lens results in the development of cataracts, indicating Chol and CBDs play a key role in preventing the development of cataracts [50]. However, despite various studies on α-crystallin binding to lens membranes and lipid vesicles [51,52,53,54,55,56,57,58,59,60,61,62,63], the interactions of α-crystallin with human lens membranes throughout the development of cortical cataract (CC) and nuclear cataract (NC) and the variation in interactions of α-crystallin with the cataractous CM and NM remains unclear.

Previous studies on the lens membrane have shown α-crystallin has hydrophobic regions that can interact with the lipid membrane [60,64], and multiple studies have reported the interactions of α-crystallin with the lens membrane are affected by the surface hydrophobicity of α-crystallin [56,65,66,67,68,69]. Relatedly, studies on synthetic lens membranes [66,68,70,71] and bovine lens lipid membranes [52,63,72] show α-crystallin can bind to the lens membrane and consequentially reduces lipid mobility in the membrane. In an infrared spectroscopy study, Tsvetkova et al. [73] found that the polar headgroup regions of the lipids composing the lens membrane strongly influence the membrane binding of α-crystallin. In agreeance, several studies on α-crystallin interactions with varying lens membranes report that α-crystallin is able to bind to the lens membrane, and these interactions are likely done via hydrophobic interactions [51,54,70,72]. Additionally, a study by Cenedella and Chandrasekher showed bovine lens membranes have a high capacity for α-crystallin binding, but intrinsic membrane proteins may impact these protein–lipid interactions [69]. Moreover, Su et al. studied the binding capacity of αA-crystallin with intrinsic protein containing bovine and human lens CM and NM and found that the interaction of αA-crystallin is associated exclusively with cellular membrane lipids [55]. To the best of our knowledge, previous studies of α-crystallin binding to lens lipid membranes were performed either with only CMs [51,54,62,72], total lens lipid extracted from the whole lens [52,63,69,74], or contained intrinsic membrane proteins [54,63,69]. Moreover, we have recently studied the binding of α-crystallin in bovine CM and NM with lipids derived from a single lens [75], but no studies have been conducted on the binding of α-crystallin in separate human CM and NM derived from a single lens.

We have previously used the Electron Paramagnetic Resonance (EPR) spin labeling method to study the binding of α-crystallin with individual lens lipids and model eye lens lipid membranes [66,67,68,70]. Our initial studies on α-crystallin binding to single and dual component lipid membranes showed the physical membrane properties and binding of α-crystallin is strongly modulated by lipid structure (headgroup, curvature, acyl chain length, and degree of unsaturation) [68,71]. Additional studies of α-crystallin in Chol-containing lipid membranes showed that both the membrane lipid and Chol composition strongly affects the membrane interactions of α-crystallin and the physical membrane properties [67,70]. Our most recent studies on α-crystallin interactions with isolated bovine lens cortical and nuclear total lipids showed α-crystallin binds to both the CM and NM but has significantly higher binding in the NM due to the variations in lipid and Chol composition between the lens regions [75]. Ultimately, these studies show α-crystallin binds to the lens lipid membrane and is strongly modulated by the lens membrane lipid and Chol composition. To build on these previous studies, we isolated the cortical and nuclear total lipids from single human lenses and applied the EPR-spin labeling method to analyze the interactions of α-crystallin with human CM and NM throughout the development and progression of NC and CC. By analyzing separate NM and CM from a single human lens, we can analyze the variation in interactions both in separate regions of the eye lens and at differing cataract grades to see how the lens changes found throughout cataract development influences the membrane’s physical properties (mobility, order, and hydrophobicity) and the binding of α-crystallin to the membranes. In addition to analyzing the changes in interactions throughout cataract development, by analyzing single lenses, factors such as age, gender, race, and health history can be accounted for to gather more insight into how different risk factors and causes of cataracts may further influence the lens lipid and Chol composition and, consequentially, α-crystallin membrane binding. Therefore, it is essential to study the binding of α-crystallin to human lens CM and NM made of total lipids (lipid plus Chol) derived from single human lenses without intrinsic membrane proteins to better understand the interactions of α-crystallin with the eye lens lipid membrane throughout the progression of cataracts. Furthermore, this capability verifies the feasibility of performing experiments with single human lenses, allowing for a more precise analysis of α-crystallin membrane binding in human eye lenses and cataract development in eye lens studies. 

## 2. Results

### 2.1. MSO by α-Crystallin in Individual Eye Lens Cortical and Nuclear Membranes with Varying Cataract Grade

**Figure 1:**
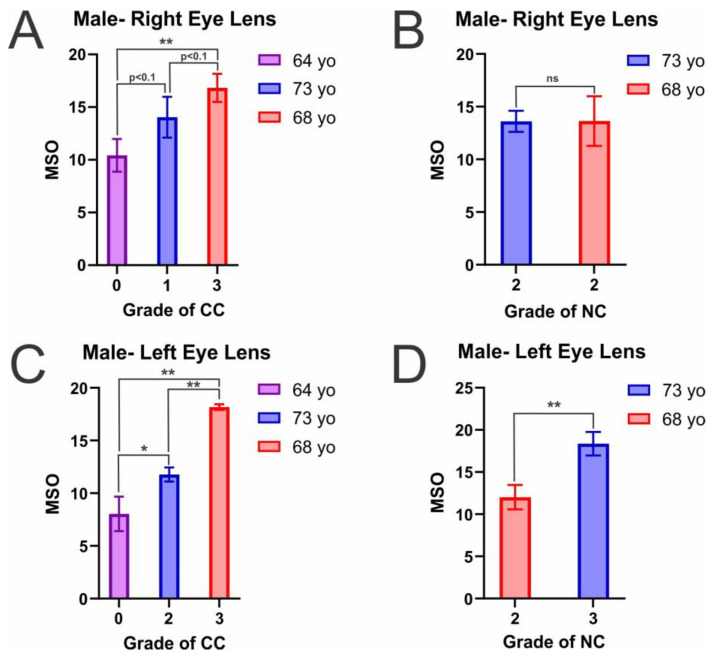
The percentage of membrane surface occupied (MSO) by α-crystallin plotted as a function of cataract grade for male lens CMs and NMs incubated with 23.06 µM of α-crystallin. (**A**) The plot of MSO by α-crystallin in CMs isolated from the male right eye lens cortex (64, 68, and 73 yo) with an increase in CC grade. (**B**) The plot of MSO by α-crystallin in NMs isolated from the male right eye lens nucleus (68 and 73 yo) with the same NC grade, regardless of age. (**C**) The plot of MSO by α-crystallin in CMs isolated from the male left eye lens cortex (64, 68, and 73 yo) with an increase in CC grade. (**D**) The plot of MSO by α-crystallin in NMs isolated from the male left eye lens nucleus (68 and 73 yo) with an increase in NC grade. The results are the mean ± standard deviation (σ) from at least three independent experiments. * and ** represent a *p*-value < 0.05 and <0.01, respectively. Changes with a *p*-value between 0.05 and 0.1 are depicted as *p* < 0.1, and “ns” represents not significant.

**Figure 2:**
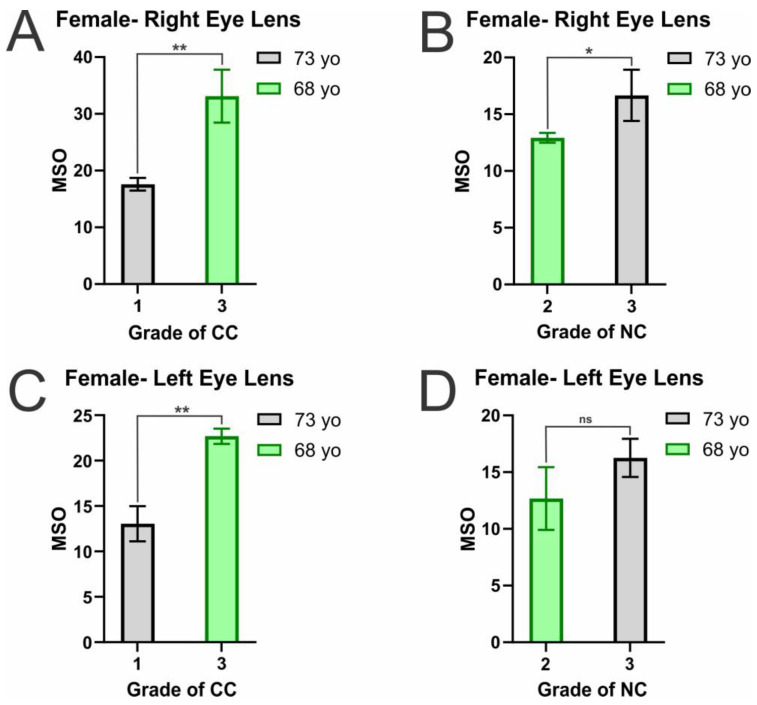
The percentage of membrane surface occupied (MSO) by α-crystallin plotted as a function of cataract grade for female lens CMs and NMs incubated with 23.036 µM of α-crystallin. (**A**) The plot of MSO by α-crystallin in CMs isolated from the female right eye lens cortex (68 and 73 yo) with an increase in CC grade. (**B**) The plot of MSO by α-crystallin in NMs isolated from the female left eye lens nucleus (68 and 73 yo) with an increase in NC grade. (**C**) The plot of MSO by α-crystallin in CMs isolated from the female left eye lens cortex (68 and 73 yo) with an increase in CC grade. (**D**) The plot of MSO by α-crystallin in NMs isolated from the female left eye lens nucleus (68 and 73 yo) with an increase in NC grade. The results are the mean ± standard deviation (σ) from at least three independent experiments. * and ** represent a *p*-value < 0.05 and <0.01, respectively. “ns” represents not significant.

As shown in Figure 1A–D, the percentage of membrane surface occupied (MSO) by α-crystallin in the male lens CM and NM displayed a positive relationship with the grade of cataracts. Therefore, with an increase in male CC (Figure 1A,C) and NC (Figure 1B,D) grade, there is a corresponding increase in the MSO by α-crystallin. This increase in MSO with the development of cataracts is further displayed in Table 1 and is statistically significant (*p* ≤ 0.05) across all male NMs and in four of six CMs. However, the male CMs found not to be significant with a *p* ≤ 0.05 were both in the right eye lens CM (Figure 2A), in which the MSO increase from the grade 0 (64-year-old (yo)) to the grade 1 CC (73 yo) donors and from the grade 1 CC (73 yo) to the grade 3 CC donor (68 yo) were both significant with a *p* < 0.1, having *p* values of 0.054079 and 0.084199, respectively. Therefore, while the increase in MSO by α-crystallin is less pronounced in these male CMs, there is still an apparent increase in MSO with an increase in CC grade. Moreover, amongst this set of CMs, the increase in MSO between the lowest (0) and highest (3) CC grades is firmly statistically significant (*p* = 0.005703). Therefore, while there is variance in the data, in both the CM and NM, an increase in cataract grade allows for increased α-crystallin membrane binding. This data indicates the changes in lipid and Chol composition and the reduction in the amount and size of CBDs, seen with the development of cataracts, alters the lens membrane environment, leading to increased α-crystallin membrane binding in both the male lens CM and NM.

Furthermore, Figure 1B shows the MSO by α-crystallin in the male right eye lens NM from donors with different ages but the same NC grade of 2. Between these two donors (68 and 73 yo), there was no significant difference (*p* = 0.495507) in MSO, indicating age-related lens changes by about five years do not seem to play a substantial role in MSO but is more dependent on cataract-associated changes. This notion that MSO is more affected by cataract grade is likely due to the major changes in lipid and Chol composition seen with the development of cataracts. Previous studies have found that with the development of cataracts, human eye lenses increase in SL content while decreasing in Chol content, reducing the size and amount of CBDs in CM and NM [42,43,53]. Moreover, we have previously shown that α-crystallin has the highest binding levels in sphingomyelin (SM)-predominant membranes, but adding Chol can interrupt and inhibit α-crystallin binding [70]. Additionally, the male 68 yo donor underwent radiation therapy, which has been shown to be directly linked to the development of cataracts and may explain why this donor has the highest CC grade and, consequentially, the highest MSO by α-crystallin in the CM. Relatedly, the 73 yo male donor was both a former smoker and had been diagnosed with Chronic Obstructive Pulmonary Disorder (COPD); however, the donor health history information did not indicate what medications the donor took for COPD treatment. Treatment with one of the primary treatments i.e., inhaled corticosteroid (ICSs), has also been associated with cataract development [76,77,78,79,80,81,82,83,84,85,86], which could explain why this donor showed the highest NC grade and in turn the most binding of α-crystallin to the NM. Additionally, all of these health conditions have been shown to cause oxidative stress, which can promote cataractogenesis and reduce the lens membrane Chol content [35,44,45,46,47,48], in turn reducing the size and amount of CBDs in the CM and NM [43], which is likely why there is an increase in MSO by α-crystallin in these donors with increases in cataract grade. Therefore, as the loss of Chol is more uniquely associated with oxidation and the development of cataracts, it is likely a critical factor in modulating α-crystallin binding to the lens membrane and a primary reason why α-crystallin binding increases with the development of both CC and NC.

The MSO by α-crystallin in female CM and NM is shown in Figure 2A–D. Identical to the trend seen in males, the MSO by α-crystallin in female CM and NMs showed a positive relationship with cataract grade. This increase in MSO with an increase in cataract grade is statistically significant, with a *p* ≤ 0.05 across all female CM and right eye lens NM. However, while there still appears to be an increase, the difference in MSO between the female left lens grade 2 (68 yo) and grade 3 NC (73 yo) donors was not statistically significant (*p* = 0.146177). The lack of significance is likely due to the similarity in NC grade between the two donors. While the difference between the right eye lens NC grade 2 and 3 was statistically significant (*p* ≤ 0.05), the difference was still less pronounced than in the MSO difference seen between grade 1 and 3 CC, indicating the more extensive the change in cataract grade, the larger the increases in α-crystallin membrane binding. This trend is similar to that seen in males, in which increased cataract grade resulted in increased MSO, but the increase between similar cataract grades was not as pronounced. Therefore, while the lipid and Chol composition alterations are likely causing these increases in MSO, the variation may differ with individuals and be less pronounced between similar cataract grades.

Ultimately, this data shows that in both male (Figure 1A–D) and female (Figure 2A–D) eye lens CM and NM, with the development and progression of CC and NC, the lens alterations allow for increased MSO by α-crystallin. This increase in MSO is likely due to the changes in the CM and NM lipid, Chol, and CBD composition seen with the development and progression of cataracts. As previously discussed, human eye lens membranes, with aging, increase in SM and Chol content significantly, and with the development of cataracts, the Chol content is reduced, reducing the size and amount of CBDs in the CM and NM [42,43,53]. Interestingly, CM and NM cataract grade-matched male and female donors have similar α-crystallin binding levels. In both males and females, there were donors with CC grades of 1 and 3 and NC grades of 2 and 3. As shown in Table 1, in the NM, males and females have very similar MSO, with both genders having an MSO around 13% and 17% for grade 2 and 3 NC grades, respectively. Moreover, in grade 1 CC, both genders showed binding levels around 15% MSO. Interestingly, in CC grade 3, both genders showed the highest MSO by α-crystallin; however, there is a deviation from the trend, with males having an average MSO around 18% while females had an average MSO around 28%. Therefore, it appears the initial development and progression of both CC and NC have similar characteristics in men and women, but the progression begins to differ and becomes more pronounced in females with late-stage CC. Studies have found that females are more likely to develop cataracts [87,88], and being female is now considered a significant risk factor for developing both CC and NC [89]. Therefore, this difference in gender may play a role in increasing female susceptibility towards cataracts and could be a reason for the late-stage MSO levels in female eye lenses being more pronounced than those found in male lenses. Moreover, the 68 yo female donor with the highest MSO by α-crystallin in the CM was a long-term smoker, pre-diabetic, and had hypertension, which have all been associated with increasing oxidative stress and the development of cataracts [77,78,79,80,81,82,83,84,85,86,90,91,92,93,94,95,96,97,98,99,100,101,102,103,104,105,106,107,108,109,110,111,112]. As oxidative stress has been shown to likely reduce the lens Chol composition [35,44,45,46,47,48], and reduced Chol is shown to lead to a diminished number and size of CBDs in CM and NM [43], the development of cataracts [50], and allow for increased α-crystallin binding [67,70,75], a reduction in Chol and CBDs content is likely why the highest MSO is seen in this donor. Therefore, in unison with gender-related risks, these health conditions will likely reduce the Chol and CBD content and allow for increased membrane binding with an increase in cataract grade.

### 2.2. Membrane Mobility on the Surface of Individual Eye Lens Cortical and Nuclear Membranes with α-Crystallin Binding

**Figure 3:**
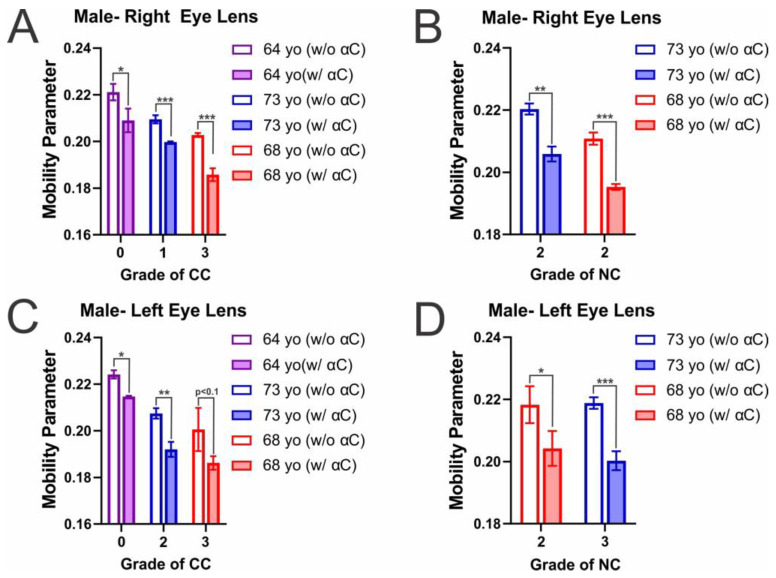
The mobility parameter plotted as a function of individual cataract grade with (w/) and without (w/o) α-crystallin (αC) in isolated male lens CM and NM. (**A**) The membrane mobility parameter with (shaded) and without (clear) α-crystallin in CMs isolated from the male right eye lens cortex (64, 68, and 73 yo) with an increase in CC grade. (**B**) The male mobility parameter with (shaded) and without (clear) α-crystallin in NMs isolated from male right eye lens nucleus (68 and 73 yo) with the same NC grade. (**C**) The membrane mobility parameter with (shaded) and without (clear) α-crystallin in CMs isolated from the male left eye lens cortex (64, 68, and 73 yo) with an increase in CC grade. (**D**) The plot of mobility parameter with (shaded) and without (clear) α-crystallin in NMs isolated from the male left eye lens nucleus (68 and 73 yo) with an increase in NC grade. The results are the mean ± standard deviation (σ) from at least three independent experiments. *, **, and *** represent a *p*-value <0.05, <0.01, and <0.001. Changes with a *p*-value between 0.05 and 0.1 are depicted as *p* < 0.1.

**Figure 4:**
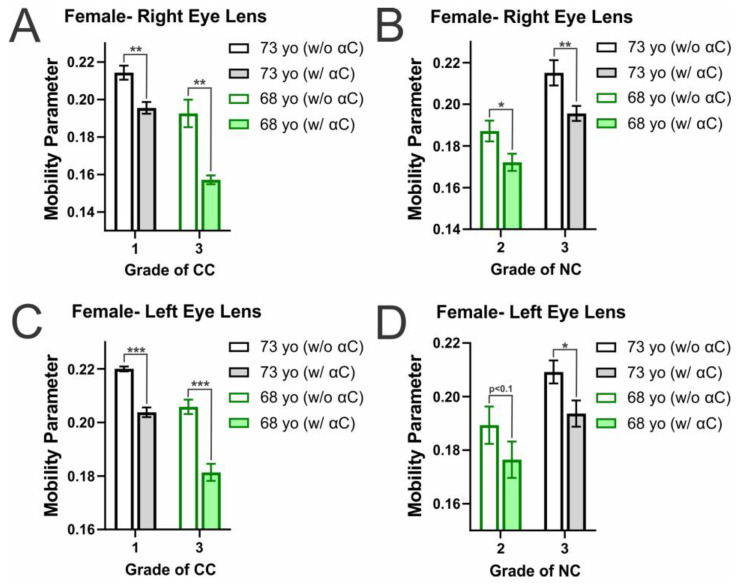
The mobility parameter plotted as a function of individual cataract grade with (w/) and without (w/o) α-crystallin (αC) in isolated female lens CM and NM. (**A**) The membrane mobility parameter with (shaded) and without (clear) α-crystallin in female right eye lens CMs (68 and 73 yo) with an increase in CC grade. (**B**) The female right eye lens NM (68 and 73 yo) mobility parameter with (shaded) and without (clear) α-crystallin with an increase in NC grade. (**C**) The membrane mobility parameter with (shaded) and without (clear) α-crystallin in CMs isolated from the female left eye lens cortex (68 and 73 yo) with an increase in CC grade. (**D**) The plot of mobility parameter with (shaded) and without (clear) α-crystallin in NMs isolated from the female left eye lens nucleus (68 and 73 yo) with an increase in NC grade. The results are the mean ± standard deviation (σ) from at least three independent experiments. *, **, and *** represent a *p*-value < 0.05, <0.01, and <0.001, respectively. Changes with a *p*-value between 0.05 and 0.1 are depicted as *p* < 0.1.

Shown in Figure 3A–D are the mobility parameters, a measure of mobility at the surface of the eye lens lipid membrane [66,68] for the male CM and NM with increasing cataract grade. In both the male CM and NM, the binding of α-crystallin at the membrane surface decreases membrane mobility. As additionally shown in Table 2, this decrease in mobility with α-crystallin binding is statistically significant, with a *p* ≤ 0.05 in all male NM and all except one CM. The only exception is in the left eye lens (Figure 4C), in which the grade 3 CC (68 yo) still showed a decrease in mobility with α-crystallin binding that was significant with a *p* < 0.1 (*p* = 0.065186). In addition to α-crystallin binding reducing mobility in the CM, the mobility parameter between α-crystallin absent controls decreases with an increase in CC grade. This decrease in mobility with an increase in CC grade is statistically significant, with a *p* ≤ 0.05 in all right eye lens CM but not in all male left eye lens CM. In the male left lens CM, the change in mobility was not significant between the CC grade 0 (64 yo) and CC grade 3 (68 yo) (*p* = 0.064831) and between CC Grade 2 (73 yo) and CC grade 3 (68 yo) donors (*p* = 0.204935). However, while the change is not statistically significant, the change between the CC grade 0 and CC grade 3 is significant with a *p* < 0.1, and is much smaller than the *p*-value of the CC grade 2 vs grade 3. This indicates that while there may be variation, there is a more considerable difference in mobility between male donors with a larger difference in CC grade. Additionally, the mobility parameter in male NM does not appear to follow a clear trend, with there being no significant difference between controls of NC grade 2 (68 yo) and NC grade 3 (73 yo) (*p* = 0.453542). However, the mobility between NC grade 2 (68 yo) and NC grade 2 (73 yo) is significantly different (*p* ≤ 0.05) between individuals with the same NC grade but different ages, showing that lipid mobility may also vary with the individual and be affected by age-related changes such as the change in lipid and Chol composition. 

In our previous studies on model membranes composed from a mixture of the four major eye lens lipids (i.e., SM, PC, phosphatidylserine (PS), and phosphatidylethanolamine (PE)) and Chol, we have found that an increase in SM content decreases the mobility near the membrane surface [70,113]. Interestingly, in the presence of Chol, the model membranes with high SM content have lower mobility on the membrane surface when compared with model membranes of varying lipid (SM, PC, PS, and PE) composition and decreasing the Chol content in the membranes increases the mobility on the membrane surface [70,113]. Therefore, the increase in SM content found in cataractous lenses [114] and the decrease in Chol and CBD content found in cataractous lenses [43,115] has the opposite effect on mobility near the membrane surface; this may be the reason for the decrease in mobility with an increase in CC grade. Moreover, the change between CMs with a grade of one CC grade separation is largest with the initial development of cataracts, but the relative difference decreases with the progression of CC. This lessening in trend may be due to the reduction in Chol content shown to occur with the progression of cataracts, as a reduction in Chol is shown to cause an increase in mobility near the membrane surface [70,71,75,113], alleviating the decrease in mobility seen from the increase in SM content. Conversely, the change between α-crystallin absent controls of the NM did not follow the trend seen in the CM, showing a slight but non-significant increase in mobility with an increase in NC grade. While cataractous lenses have been shown to increase in SM content overall, the NM is still reported to have possible reductions in SM with cataract development, resulting in lower levels of SM than in the CM, and the decrease in Chol content in cataractous lenses is significantly larger in the NM than in the CM [43,114,115]. Therefore, the reduction in SM may impact the NM, but the significant decrease in Chol and the size and amount of CBDs may have a much larger impact on the mobility of the NM, which explains the loss of trend and possible increases in mobility seen in the NM.

Further displayed in Figure 4A–D is mobility on the membrane surface for female CM and NMs, in which the binding of α-crystallin also correlates with a consequential decrease in mobility on the membrane surface. This decrease in mobility with α-crystallin binding is found to be statistically significant with a *p* ≤ 0.05 in all female CMs and all NM apart from the left lens NC grade 2 (68 yo), in which there is an apparent decrease in mobility that is statistically significant with a *p* < 0.1 (*p* = 0.067295). Therefore, the binding of α-crystallin decreases mobility at the CM and NM surface. Moreover, similar to that seen in the male mobility data (Figure 3A,C), the mobility in the female CM were all shown to significantly decrease with an increase in CC grade (*p* ≤ 0.05). This data indicates that the changes in lipid and Chol composition seen with the development of CC reduces mobility at the membrane surface. However, differing from the males, the mobility parameter in both female left and right NMs significantly increases with an increase in NC grade (*p* ≤ 0.05). As previously discussed with the development of cataracts, there is generally an increase in SM content shown to cause a decrease in membrane mobility, while there is also a decrease in Chol and CBD content, causing an increase in membrane mobility [42,53,67,70]. However, the decrease in Chol content and the decrease in size and amount of CBDs is significantly more pronounced in the cataractous NM relative to the cataractous CM [43]. Therefore, the increase in SM content found in cataractous lenses may be the reason for the decrease in mobility with an increase in CC grade, and while affected, the minor change in Chol content is not enough to counter the effects of increased SM content. Conversely, while there was no clear trend in mobility and NC grade in the male NM, the female NM showed significant increases in mobility with an increase in NC grade. This aligns with a decrease in Chol being more prominent in the NM, allowing for increased mobility at the membrane surface. Interestingly, while it is not clear why the NM trend is only pronounced in females and not males, as previously discussed, females have been shown to be more likely to develop cataracts [116,117,118,119,120], which could be a factor as to why the trends seen are again more pronounced in females relative to males. Moreover, as previously mentioned, health and lifestyle factors found in these donors, such as smoking, diabetes, hypertension, radiation therapy, and COPD, may be changing the lens environment and, consequentially, the lipid and Chol composition, which may affect the mobility data seen for each donor.

This data aligns with the previously discussed MSO data for males (Figure 1A–D) and females (Figure 2A–D), in which the decrease in mobility parameter between controls and their α-crystallin containing counterpart was generally more considerable with an increase in MSO. The only two exceptions for this trend were both found in the male CM in which the decrease in the right eye CM was slightly less in the CC grade 1 compared to the CC grade 0 and in the left eye lens CM, where the decrease seen in the CC grade 3 was slightly smaller than that seen in the CC grade 2. However, in all other male and all female donors, the decrease in mobility parameter with α-crystallin binding was increased with a corresponding increased MSO. Furthermore, the two largest reductions in MSO were seen in the 68 yo female left and right eye CM, which also displayed the highest MSO values. These results, therefore, indicate that α-crystallin binding at the membrane reduces mobility at the membrane surface and, generally, with increased binding found with an increase in both CC and NC, there is a larger impairment of membrane mobility on the surface.

### 2.3. Membrane Order near the Surface of Individual Eye Lens Cortical and Nuclear Membranes with α-Crystallin Binding

**Figure 5:**
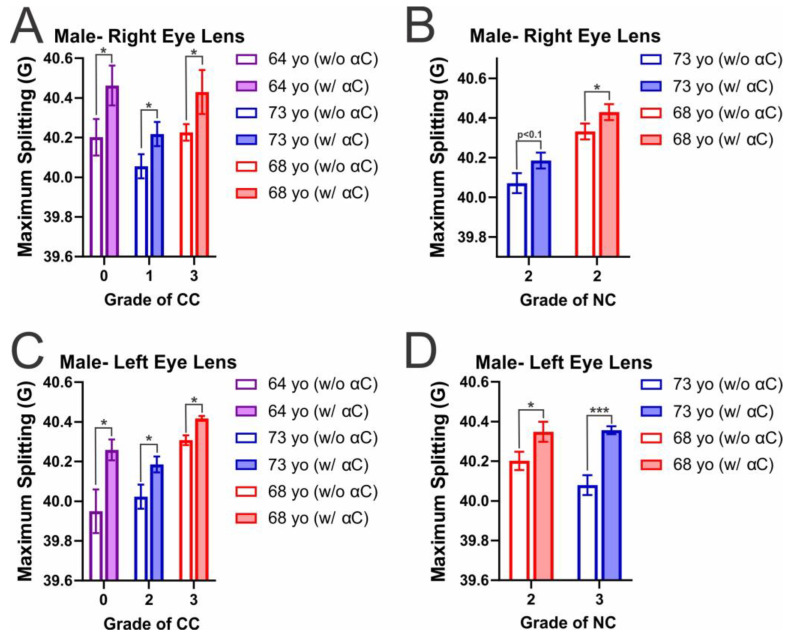
The maximum splitting (membrane order near the surface) plotted as a function of individual cataract grade with (w/) and without (w/o) α-crystallin (αC) in isolated male lens CM and NM. (**A**) The membrane order with (shaded) and without (clear) α-crystallin in CMs isolated from the male right eye lens cortex (64, 68, and 73 yo) with an increase in CC grade. (**B**) The maximum splitting values with (shaded) and without (clear) α-crystallin in NMs isolated from male right eye lens nucleus (68 and 73 yo) with the same NC grade. (**C**) The membrane order in male left eye lens CMs with (shaded) and without (clear) α-crystallin (64, 68, and 73 yo) with an increase in CC grade. (**D**) The membrane order with (shaded) and without (clear) α-crystallin in NMs isolated from the male left eye lens nucleus (68 and 73 yo) with an increase in NC grade. The results are the mean ± standard deviation (σ) from at least three independent experiments. *, and *** represent a *p*-value < 0.05 and <0.001, respectively. Changes with a *p*-value between 0.05 and 0.1 are depicted as *p* < 0.1.

Figure 5A–D shows the maximum splitting values, which is a measure of order at the membrane surface for the male CM and NM. The addition of α-crystallin with both male CM and NM increased maximum splitting, meaning the binding of α-crystallin causes an increase in order at the membrane surface. This increase in membrane order was found to be statistically significant (*p* ≤ 0.05) amongst all CMs and in all but one left lens NM (Figure 5B), in which NC grade 2 (73 yo) showed an increase in maximum splitting that was still significant with a *p* < 0.1 (*p* = 0.052696). Unlike the mobility parameter data, the membrane order without α-crystallin does not appear to follow any clear trends. However, the membrane order without α-crystallin significantly differs (*p* ≤ 0.05) between donors with the same cataract grade. Therefore, as the development of CC and NC as well as an increase in cataract grade is not shown to have a clear impact on membrane order, and individuals of different ages with the same cataract grade can have significant differences, it appears factors like age and previously discussed individual health history may play a key role in altering membrane order at the CM and NM surface.

**Figure 6:**
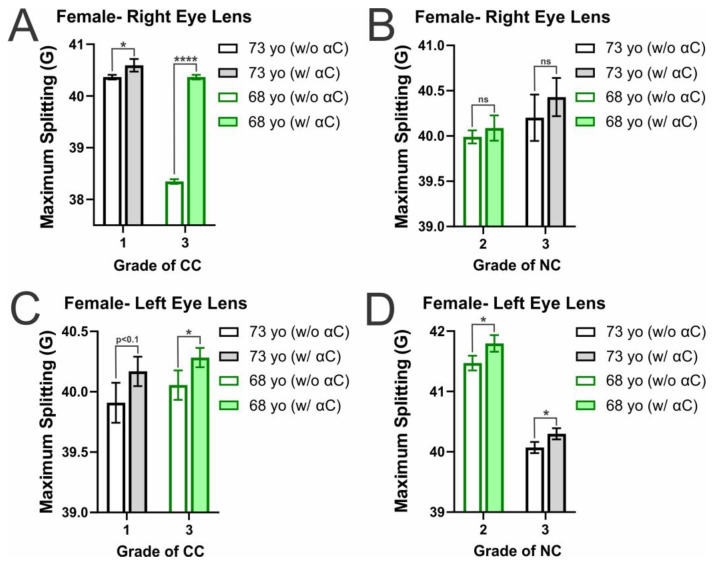
The maximum splitting values plotted as a function of individual cataract grade with (w/) and without (w/o) α-crystallin (αC) in isolated female lens CM and NM. (**A**) The membrane order with (shaded) and without (clear) α-crystallin in female right eye lens CMs (68 and 73 yo) with an increase in CC grade. (**B**) The maximum splitting values for the female right eye lens NMs (68 and 73 yo) with (shaded) and without (clear) α- crystallin with an increase in NC grade. (**C**) The membrane order with (shaded) and without (clear) α-crystallin in CMs isolated from the female left eye lens cortex (68 and 73 yo) with an increase in CC grade. (**D**) The membrane order with (shaded) and without (clear) α-crystallin in NMs isolated from the female left eye lens nucleus (68 and 73 yo) with an increase in NC grade. The results are the mean ± standard deviation (σ) from at least three independent experiments. * and **** represent a *p*-value < 0.05 and <0.0001, respectively. Changes with a *p*-value between 0.05 and 0.1 are depicted as *p* < 0.1, and “ns” represents not significant.

Figure 6A–D shows maximum splitting data for the female CM and NM in which α-crystallin membrane binding increased membrane order at the membrane surface. While all CM and NM showed some increase in order with α-crystallin binding, the variance in membrane order was found to be statistically significant (*p* ≤ 0.05) amongst all CCs except in the left lens CC grade 1 (73 yo), which had a *p*-value of 0.07405 making the increase in membrane order significant with a *p* < 0.1. Moreover, in the female left lens NM, the addition of α-crystallin resulted in significant (*p* ≤ 0.05) increases in membrane order; however, in the female right lens NM, the change with both NC grade 2 (68 yo) and NC grade 3 (73 yo) is shown to increase in maximum splitting but was not statistically significant with *p* values of 0.267131 and 0.193337, respectively. Therefore, while α-crystallin binding is shown to cause an increase in membrane order near the surface, the increase varies between different donors, with the most significant change in mobility being found in the 68 yo female right eye CM. Additionally, in the left eye CM, this donor also showed the most significant increase in order with α-crystallin binding, and as previously mentioned, this donor had prediabetes, hypertension, and was a long-term smoker, which can all increase oxidation; this in turn reduces Chol content [35,44,45,46,47,48], which reduces the size and amount of CBDs in membranes [43], altering the lens membrane environment and allowing for increased α-crystallin binding, which is likely a major factor as to why the largest increases in membrane order are found in this donor.

Interestingly, in males and females, the CM was most impacted by α-crystallin binding with a statistically significant increase (*p* ≤ 0.05) in membrane order on the surface, with only one out of ten combined CMs not showing a statistically significant increase. However, while α-crystallin binding is still shown to increase membrane order, the NM seemed less impacted, with three out of eight total donors not showing a statistically significant increase (*p* ≤ 0.05) in membrane order. Additionally, the only CM not found to be statistically significant with a *p* ≤ 0.05 was significant with a *p* < 0.1 and had a lower *p*-value (*p* = 0.07405) than two of the three non-significant NM *p* values (*p* = 0.052696, 0.193337, and 0.267131). Therefore, while affected, the NM order is shown to be less impacted with α-crystallin binding relative to the CM. In the case of maximum splitting, prior studies have shown that an increase in SM and Chol content both correlate with an increase in membrane order [26,67,70]. Therefore, the rise in SM seen with aging and the development of cataracts [42,53,114] should correlate with an increase in membrane order, but the decrease in Chol and CBDs content in cataractous lenses should simultaneously lower the membrane order. However, the trend in the NM is not clear, and the NM has been shown to have possible decreases in SM content with cataract development [43]. Therefore, the synergistic effect of changing SM content and decreasing Chol content may play a significant role in modulating NM order seen in the development of NC but requires future studies to be understood better.

### 2.4. Hydrophobicity on the Surface of Individual Eye Lens Cortical and Nuclear Membranes with α-Crystallin Binding

**Figure 7:**
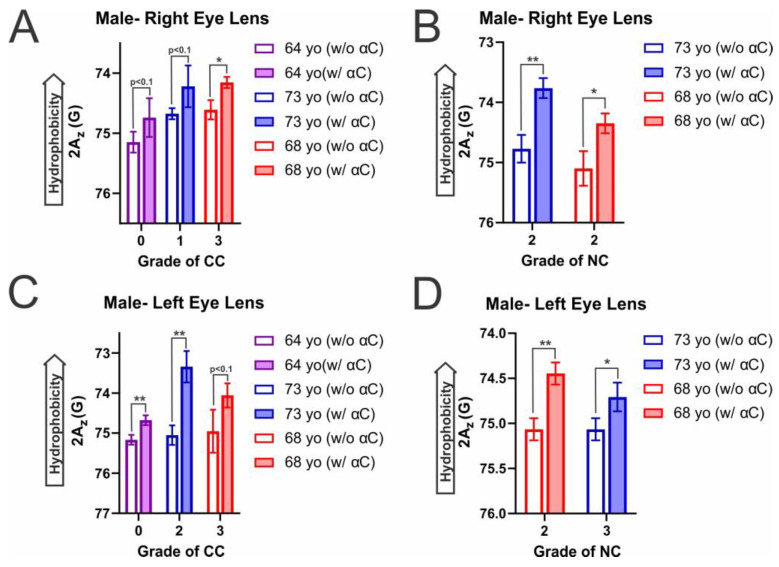
The hydrophobicity values plotted as a function of individual cataract grades with (w/) and without (w/o) α-crystallin (αC) in isolated male lens CM and NM. (**A**) The hydrophobicity values from the male right eye lens CM (64, 68, and 73 yo) with (shaded) and without (clear) α-crystallin with an increase in CC grade. (**B**) The hydrophobicity values with (shaded) and without (clear) α-crystallin in NM isolated from male right eye lens nucleus (68 and 73 yo) with the same NC grade. (**C**) The hydrophobicity with (shaded) and without (clear) α-crystallin in CMs isolated from the male left eye lens cortex (64, 68, and 73 yo) with an increase in CC grade. (**D**) The hydrophobicity data with (shaded) and without (clear) α-crystallin in NMs isolated from the male left eye lens nucleus (68 and 73 yo) with an increase in NC grade. The results are the mean ± standard deviation (σ) from at least three independent experiments. * and ** represent a *p*-value < 0.05 and <0.01, respectively. Changes with a *p*-value between 0.05 and 0.1 are depicted as *p* < 0.1.

**Figure 8:**
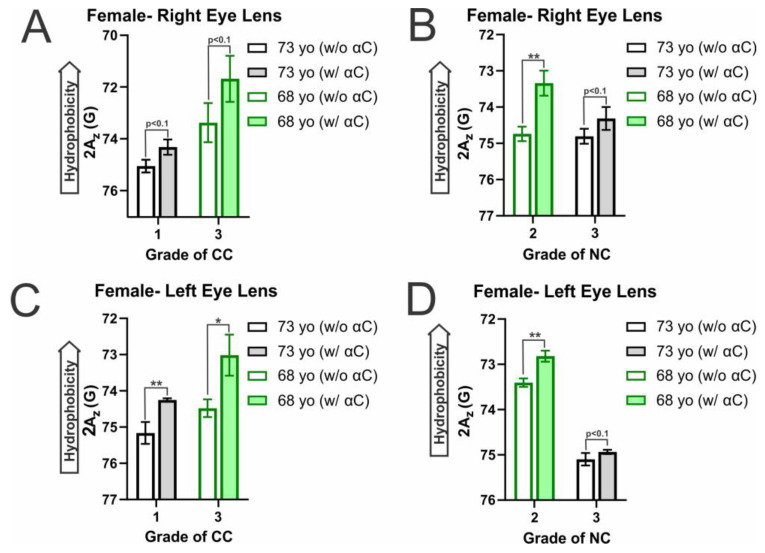
The hydrophobicity values plotted as a function of individual cataract grades with (w/) and without (w/o) α-crystallin (αC) in isolated female lens CM and NM. (**A**) Hydrophobicity values with (shaded) and without (clear) α-crystallin in CMs isolated from the female right eye lens cortex (68 and 73 yo) with an increase in CC grade. (**B**) The hydrophobicity with (shaded) and without (clear) α-crystallin in the female left eye lens NM (68 and 73 yo) with an increase in NC grade. (**C**) Hydrophobicity data from the female left eye lens CM (68 and 73 yo) with (shaded) and without (clear) α-crystallin with an increase in CC grade. (**D**) The hydrophobicity values with (shaded) and without (clear) α-crystallin from the female left eye lens NM (68 and 73 yo) with an increase in NC grade. The results are the mean ± standard deviation (σ) from at least three independent experiments. * and ** represent a *p*-value < 0.05 and <0.01, respectively. Changes with a *p*-value between 0.05 and 0.1 are depicted as *p* < 0.1.

Shown in Figure 7A–D is the hydrophobicity data for the male CM and NM samples, depicting the measured 2A_z_ value, a measure of hydrophobicity at the membrane surface. Amongst this data is a decrease in 2A_z_ value with α-crystallin binding, corresponding to an increase in hydrophobicity. This increase in hydrophobicity was seen amongst all male CM (Figure 7A,C) and was significant, with a *p* ≤ 0.05 in half of the male CM. The increases in hydrophobicity not found to be significant with a *p* ≤ 0.05 were in the male right lens CC grade 0 (64 yo) (*p* = 0.095575) and grade 1 (73 yo) (*p* = 0.073752) and the left lens grade 3 (68 yo) (*p* = 0.081356). However, these membranes still display an evident change and apparent increase in hydrophobicity with the binding of α-crystallin that are statistically significant with a *p* < 0.1. Moreover, in the NM (Figure 8B,D) with α-crystallin binding, a significant increase in hydrophobicity (*p* ≤ 0.05) was shown across all analyzed donors. Therefore, while there is variation between individuals, the binding of α-crystallin in both the male CM and NM causes an increase in hydrophobicity near the membrane surface. This increase in hydrophobicity likely implies that α-crystallin with hydrophobic residues exposed on its surface interacts with CM and NM, expelling water molecules in the headgroup regions, resulting in an increase in hydrophobicity, suggesting the hydrophobic interactions of α-crystallin with the CM and NM. The increased hydrophobicity on the surface of CM and NM forms the hydrophobic barrier for the transport of water molecules and antioxidants (glutathione), likely creating the oxidative environment inside the lens, ultimately leading to cataract formation.

Relatedly, displayed in Figure 8A–D are the membrane surface hydrophobicity levels for female CM and NM. With the addition of α-crystallin, there is an increase in hydrophobicity amongst all CM and NM samples. In the female CM, this increase was found to be statistically significant with a *p* ≤ 0.05 in all but two right lens CM (CC grade 1 (73 yo): *p* = 0.065874 and CC grade 3 (68 yo): *p* = 0.07234), which however both have increases in hydrophobicity that are significant with a *p* < 0.1. Moreover, α-crystallin binding in the female NM also increases membrane surface hydrophobicity significantly (*p* ≤ 0.05) in all but the right lens NC grade 3 (73 yo, *p* = 0.092044) and the left lens NC grade 3 (73 yo, *p* = 0.09452). Again, these membranes were still shown to increase in hydrophobicity with α-crystallin binding, and the increases were both significant with a *p* < 0.1. This data shows that α-crystallin binding in both the female CM and NM increases hydrophobicity near the membrane surface, indicating α-crystallin likely binds to the lens CM and NM via hydrophobic interactions. This agrees with our prior studies on α-crystallin interactions with model eye lens lipid membranes, where α-crystallin binding was shown to cause increases in hydrophobicity in model lens lipid membranes [70].

In addition to hydrophobicity increasing with the binding of α-crystallin, the hydrophobicity in the male and female CM, in the absence of α-crystallin, is shown to increase with increasing CC grade. While the trend is shown to persist and be significant (*p* ≤ 0.05) in most of the female samples, in males, the trend can be seen in the male right lens CM, with the increase being significant (*p* ≤ 0.05) in all but the controls of the right lens CM 73 yo (grade: 1) vs. 68 yo (grade: 3) with *p* = 0.321665 not being a significant difference. However, in the left lens CM, there was no significant difference between the three controls. In all cases that were not significant, the more different the CC grade between samples, the lower the *p*-value. Conversely, in the female NM, hydrophobicity decreased with increased NC grade. However, this decrease was only significant between controls in the female left lens NM (*p* ≤ 0.05) and was not significant between control of the right lens NM (68 yo (grade: 2) vs. 73 yo (grade: 3): *p* = 0.337249). However, this trend only appears to be present in the female NM; the same does not appear true for the male NM. In both the left and right lenses, there was no significant difference between the controls of each sample. However, only in the left eye lens is there a difference in NC grade, in which there is a slight decrease with an increase in NC grade, but the difference is not significant (*p* = 0.500).

Furthermore, both the male and female hydrophobicity aligns with the MSO data reported in Figure 1 and Figure 2A–D, as generally, the largest increases in hydrophobicity are found in the samples with the largest MSO. The only exceptions to this trend are in the left male CM, in which the change was more prominent in the CC grade 2, and in the left female NM, the change was larger in the NC grade 2 than in the grade 3 of each. However, in all other cases, the change in hydrophobicity was larger with an increase in MSO. Moreover, the previously mentioned female right eye lens CC grade 3 with the largest MSO again shows the largest increase in hydrophobicity with α-crystallin binding. This data indicates that α-crystallin binds to the membrane surface via hydrophobic interactions, leading to an increase in the relative hydrophobicity of the environment at the membrane surface.

Interestingly, the mobility parameter and hydrophobicity data follow opposite trends to varying degrees. Meaning in both the female and male CM, there is an increase in hydrophobicity, while the mobility parameter decreases with an increase in CC grade. Conversely, the NM shows possible decreases in hydrophobicity and increases in mobility parameter with an increase in NC grade. As the cataractous CM has more SM and less Chol than the cataractous NM, the increase in SM seen with cataracts may be strongly impacting the CM, while the reduction in Chol being much smaller than that seen in the NM has less of an impact on the CM mobility. Moreover, the decrease in Chol, while not apparently as impactful on membrane mobility, may contribute to the increased hydrophobicity seen with increased CC grade. Therefore, the lipid and Chol composition of the individual lenses likely modulates membrane mobility and the hydrophobic environment on the membrane surface, and with decreased mobility, there appears to be increased hydrophobicity.

## 3. Discussion

With age and cataract development, the human eye lens changes significantly in lipid and Chol composition [33,34,35,40,41,43,114,115]. Typical age-related changes in the lens lead to an increase in SM and Chol content with a consequential decrease in PC content [36,42]. However, with the development of cataracts, the lens increase in SM is exacerbated, and the lens decreases Chol and CBD content, with the decrease of Chol content being significantly more prominent in the NM relative to the CM [36,38,39,41,43,114]. While the loss of Chol in the NM is larger, the Chol content of the CM remains lower than in the NM [38,39,43]. Agreeingly, in this study, the highest α-crystallin binding levels may be seen in the CM and with increased CC and NC grade, indicating the reduced Chol and CBD content in the CM and with cataractogenesis allows for increased α-crystallin binding. This binding of α-crystallin results in a decrease in membrane mobility and an increase in order at the membrane surface. Moreover, the mobility in CM decreases with an increase in CC grade, while the NM shows possible increases in mobility with an increase in NC grade. As previously discussed, the loss of Chol increases membrane mobility, while the increase in SM generally decreases membrane mobility. Therefore, as the Chol loss is more prominent in the cataractous NM than the cataractous CM, the loss of Chol and diminishment of CBDs may be more impactful on the NM mobility, while the increase in SM may impact the CM more. Moreover, the study reported in this paper shows that α-crystallin binding to the human CM and NM increases the hydrophobicity at the surface of the membranes, indicating that such binding likely creates a hydrophobic barrier for the passage of ionic and polar molecules, including antioxidants (glutathione), creating an oxidative environment inside the lens leading to the development of cataract. Our previous studies have shown that the decreased Chol content in the bovine CM [75] and model lens lipid membranes [67,70,71] increased the hydrophobicity below the membrane’s surface and further increased the binding of α-crystallin. While similar in MSO between males, the grade 3 CC showed the highest MSO, which, in unison with the previously discussed donor health-related factors, the decreased Chol content and reduced CBD content may increase the hydrophobic environment at the membrane surface, allowing for higher levels of α-crystallin binding. However, except in the 68 yo female donor, there is no significant difference in hydrophobicity between the CM and NM of the same lens, indicating the water accessibility on the surface of these membranes is generally similar. Moreover, while the data has variance, the general trends in hydrophobicity data and mobility parameters seen with an increase in cataract grade are inverse between the NM and CM. Increases in SM and Chol both lower mobility, and while SM has a minimal effect, an increase in Chol lowers the relative hydrophobicity near the membrane surface [67,70,75,113]. Therefore, the lipid and Chol environment between the CM and NM may strongly differ, creating variations in α-crystallin interactions and impacting the individual development of NC and CC. Therefore, these lipid and Chol variances between separate lens regions and the progression of cataracts modulates the hydrophobic binding of α-crystallin to human eye lens membranes, and this binding has a positive relationship with cataract grade. Moreover, this study shows the feasibility of experiments using the total lipids extracted from a single lens cortex and nucleus of a human, which is critical to fully understand the individual lens lipid composition changes accounting for the donor health history information, sex, race, grade of cataract (CC and NC) and causes of α-crystallin binding to lens membrane leading to the development of human cataracts.

In addition to age-related changes, an individual’s medical history and lifestyle factors may alter the lipid and Chol composition of the human lens. As previously discussed, while males and females show similar trends of α-crystallin binding in both the CM and NM at varying cataract grades, a majority of experimental trends are pronounced amongst female donors. The highest MSO found amongst any of the analyzed lens CM and NM was in the female 68 yo right eye CM (CC grade 3), and interestingly, the left eye CM from the same donor displayed the second highest MSO compared to binding levels found from any other donor. Moreover, females showed more pronounced changes in mobility with increasing cataract grade, particularly in the NM, compared to males. As previously mentioned, studies have found females are more likely to develop cataracts, [87,88] making being female a significant risk factor for developing both CC and NC [89], and the development of cataracts significantly changes the Chol and lipid composition in the lens. The alteration in Chol and lipid composition changes the mobility parameter significantly in CM and NM, as reported in this study, suggesting hormonal factors might contribute to gender-based differences in the mobility near the headgroup regions of CM and NM. It has been found that women appear to be increasingly prone to cataracts due to variations in estrogen levels seen post-menopause, which is likely a primary reason for the increased trends seen in females. The eye lens has been shown to have estrogen receptors, and the knockout of these receptors has been shown to lead to the development of cataracts in vivo [120]. Relatedly, other studies have found estrogen to have protective effects against the development of cataracts [116,117,118,119,120], with estrogen showing to have antioxidative functions [121]. These antioxidative properties are believed to be uniquely important in protecting against ROS in the lens [121], which could prevent cataract-inducing lens changes like lipid oxidation. As mentioned, lipid oxidation can alter the lens lipid and Chol composition [35,44,45,46,47,48], which strongly modulates α-crystallin binding and the physical properties of the membrane [67,70]. The variation in estrogen seen in menopause may, therefore, play a role in increasing female susceptibility towards cataracts and could be a reason for the variances in mobility parameter and late-stage MSO levels found in the female eye lenses to be more pronounced than those found in male lenses. Therefore, it appears the eye lens can interact with estrogen, which has been shown to give estrogen possible protective effects against ROS and cataractogenesis. In addition to the possible role of estrogen increasing the likelihood of women developing cataracts being a reason for the pronounced trends seen, other lifestyle and health factors may play a role or work in a synergistic way to contribute to the variation in trends.

Lifestyle factors such as smoking and tobacco use have been shown to be associated with cataract development. While there is some conflicting data, smoking and tobacco use has been directly linked to an increased risk of cataracts [77,78,79,122]. Some previous studies report smoking increases the risk of developing NC but not CC [80,81,82], while others report smoking cigarettes to be associated with the development of CC [83,84,85,86]. Moreover, Raju et al. [78] found using tobacco via smoking or in a non-smokable form causes an increased risk of NC, but the use of smokeless tobacco has a stronger impact on cataract development. Therefore, while there is debate on whether tobacco itself or smoking has more of an effect on cataract development and the type of cataract, in all studies, the use of tobacco and smoking was associated with an increased risk of developing some form of cataracts. While the mechanism by which smoking leads to cataractogenesis is not known, there are several believed possible reasons for smoking increasing the risk of cataracts. A primary belief is that smoking promotes lens oxidation by directly increasing free radical activity [93] or depleting antioxidant levels [123,124,125]. Secondly, tobacco use can lead to the accumulation of heavy metals [126,127] and toxic chemicals [128,129,130] that can accumulate in the eye lenses of smokers, further reducing antioxidant availability and possibly modifying lens proteins, ultimately promoting the development of cataracts. As previously discussed, oxidation has been shown to truncate the lens lipids and force Chol out of the membrane [35,44,45,46,47,48]. This loss of Chol further reduces the size and amount of CBDs in membranes, consequentially allowing for increased α-crystallin binding and promoting cataractogenesis [26]. Therefore, the increased oxidation with smoking may be a critical factor in reducing the lens Chol content, allowing for increasing α-crystallin binding and ultimately promoting the development of cataracts. In this study, two of the donors were smokers (68 yo female and 73 yo male); however, smoking cessation has shown to significantly reduce the odds of cataractogenesis following quitting [79], and the 68 yo female with the most pronounced experimental trends was the only long-term smoker. Therefore, while the results found from both donors may be affected by smoking with an increased likelihood of lipid oxidation, the 68 yo female is likely to be the most impacted. Moreover, the 68 yo female donor showed the largest MSO levels in both the left and right lens and had the most pronounced physical properties of membrane changes relative to all other donors. Therefore, the effect of smoking on increasing oxidation levels in the eye lens may be a primary factor in both the development of cataracts and be contributing to the pronounced α-crystallin membrane interactions due to the loss of lens Chol and CBD content.

Relatedly, smoking has shown to be directly associated with the development of Chronic Obstructive Pulmonary Disorder (COPD). In this study, the 73 yo male donor was diagnosed with COPD, and while the health history information did not state what medications the donor took for treatment, inhaled corticosteroids (ICSs) are one of the primary treatments for COPD [131,132,133]. Moreover, COPD itself has not been directly linked with the development of cataracts, but treatment with ICSs has been shown to potentially be associated with cataractogenesis. Previous studies regarding the long-term effects of ICSs on the development of cataracts present conflicting results, with some studies reporting ICS use to be associated with an increased risk of cataracts [76], while other studies show there to be no clear association between the two [133,134]. Therefore, while the effects of ICSs on the development of cataracts are not apparent and require future research, the treatment of COPD may affect the lipid composition of the eye lens, leading to the development of cataracts and may be a contributing factor to the development of cataracts in the 73 yo male donor in this study. Regardless, smoking cigarettes has been shown to lead to increased risk of both COPD [79] and cataracts, meaning both the use of cigarettes and possibly the treatment for smoking-related diseases may cause changes in the lens environment and the lipid and Chol composition of the lens, leading to the development of cataracts.

Additionally, health-related issues such as diabetes and hypertension have been shown to be associated with an increased occurrence of cataracts [135,136]. Previous studies have found both diabetes and prediabetes to be related to the development of cataracts, with prediabetes resulting in the same effects but less severe than the lens changes seen with diabetes [90]. In agreeance, multiple studies report cataract formation to occur more frequently amongst diabetic patients relative to the rates of cataractogenesis in nondiabetic patients [91,92,93]. While the exact cause of diabetic cataracts is unknown, there are several possible mechanisms in which diabetes is believed to promote cataract development. Amongst these links, the lens glucose metabolism, done using the sorbitol pathway, is shown to be heavily involved in the development of diabetic cataracts. Multiple studies report a primary enzyme of the sorbitol pathway, aldose reductase, to be possibly linked to the development of diabetic cataracts [94,95], with increased aldose reductase expression showing increased the risk of cataract development in diabetic patients [97]. Moreover, the sorbitol pathway can induce oxidative stress as it both produces superoxide anions as a byproduct and uses NADPH, in turn depleting the availability of NADPH for the production of glutathione, a primary lens antioxidant [103,104]. This increased oxidative stress and loss of antioxidants consequentially creates the oxidative environment inside the lens, promoting lipid peroxidation, which likely pushes Chol out of the lens membrane, reducing the lens Chol content, diminishing the amount and size of CBDs, and consequentially allowing for increased α-crystallin lens membrane binding. Moreover, dysregulation of the sorbitol pathway can result in fluid accumulation within the lens that causes liquefication and degeneration of the lens fibers [95,98,99]. Separate from the sorbitol pathway, there has also shown to be increased expression of pro-inflammatory cytokines within the lens of diabetic patients that is believed to possibly cause cellular dysfunction and apoptosis [103,104,137]. This disruption of cellular function may further disrupt the lens homeostasis, promoting the development of cataracts. Therefore, for a multitude of possible reasons, diabetes and prediabetes are shown to cause an increased risk of cataract development and, therefore, may uniquely alter the lipid and Chol composition and change the interactions of α-crystallin with the membrane. The 73 yo female with grade 3 NC previously discussed associated with smoking that shows the most pronounced binding was also pre-diabetic. Also, a 68-year female donor with grade 3 CC associated with long-term smoking, pre-diabetic, and hypertension showed the largest MSO by α-crystallin in CM from both the left and right lens and had the most pronounced mobility changes relative to all other analyzed samples. Therefore, while the individual contribution of all the risk factors is unknown and requires future studies, being pre-diabetic and smoking paired with the risk factor of being female and being a long-term smoker, pre-diabetic, and hypertension paired with a risk factor of being female may have a synergistic effect on the alteration of lipids and reducing the lens Chol composition, explaining why the trends may be significantly more pronounced in this donor. The findings of this study suggest that managing risk factors like diabetes and changing lifestyle factors like, for example, quitting smoking might have less effect on lens oxidation (lens lipid and Chol oxidation) and thus less impact on decreasing Chol content in the fiber cell plasma membrane of the eye lens that might consequently decrease α-crystallin binding to lens membrane and prevent the development and progression of cataracts.

Relatedly, hypertension or high blood pressure has also been reported to be associated with the development of cataracts. Hypertension can increase inflammatory cytokine expression, causing increased inflammation and, in turn, disrupting lens homeostasis [102,103] and may induce conformational changes in the crystallin proteins, leading to the development of cataracts [138]. Multiple studies report people with increased severity and duration of hypertension have an increased risk of cataract development [105,106,107,108]. Additionally, while not all medications are linked, the use of select anti-hypertension medication is linked to cataract development. Specifically, studies have found that the use of either potassium-sparing diuretics [109] or beta-blockers [104,110,111,112] can alter the lens fiber membrane and the lens proteins, promoting the formation of cataracts. In this study, both the 68 yo and 73 yo females were diagnosed with hypertension. Therefore, both hypertension and the treatment of hypertension may contribute to the alteration of the lens lipid and Chol composition, leading to the development of cataracts.

In addition to hypertension medication being associated with cataracts, other treatments, such as radiation therapy and radiation exposure, have shown to be strongly associated with the development of cataracts [139,140,141]. Previous studies have found the lens is more sensitive to radiation than most other tissues [142], making the development of cataracts after radiation therapy very common. Radiation-associated cataracts generally develop between 2 and 3 years following treatment and are shown to be dose-dependent, with increased doses showing an increased likelihood of cataractogenesis [141]. While the exact mechanism of how radiation therapy causes cataracts is unclear, it is believed that radiation exposure may cause oxidative stress, abnormal cell proliferation, and mutate the crystallin proteins, all contributing to the disruption of lens homeostasis and promoting cataractogenesis [142,143,144]. While all these factors may be contributing to the development of cataracts, as previously discussed, the increase in oxidative stress likely causes a reduction in the lens Chol content and diminishes the amount and size of CBDs, which may be a primary reason for the increase in MSO by α-crystallin with the development of cataracts. In this study, the 68 yo male donor underwent radiation therapy for the treatment of cancer. While many other factors may contribute, this individual, despite being five years younger than the oldest male donor, had the highest CC grade of all the male donors and the second most progressed NC. Therefore, in unison with other health factors, this individual’s exposure to radiation therapy may significantly alter the lens lipid and Chol composition, which may be a reason for the development of late-stage CC at a relatively younger age in this donor.

The increased MSO by α-crystallin on the surface of the CM and NM with an increase in cataract grade shows both that α-crystallin binds to the human eye lens membrane and the changes in the membrane lipid, Chol and CBD composition seen throughout the progression of cataracts leads to increased α-crystallin binding. In our previous studies on the CM and NM of non-cataractous bovine lenses, we found that α-crystallin binds more to the surface of the membranes than below the membrane’s surface near the headgroup region. However, in low Chol model bovine membranes, α-crystallin penetrated the membrane surface, causing significant increases in hydrophobicity near the membrane headgroup region [75]. Agreeingly, in our previous studies on α-crystallin interactions with Chol-containing synthetic lipid membranes, we found that α-crystallin membrane binding significantly increases hydrophobicity near the membrane headgroup region, leading to the formation of a hydrophobic barrier [70,71]. These studies all show Chol may play a key role in maintaining lens homeostasis and regulating α-crystallin membrane binding, preventing the formation of a hydrophobic barrier and cataracts. Relatedly, in the study reported in this paper, the decreased Chol content and reduction in the amount and size of CBD found in lenses with an increase in cataract grade is likely a primary reason for the increased binding of α-crystallin to the NM and CM surface. At varying levels, this binding of α-crystallin is also shown to cause increases in hydrophobicity, which indicates that it may contribute to developing a hydrophobic barrier near the membrane surface. Moreover, the increased α-crystallin binding seen with increased cataract grade may allow α-crystallin to penetrate below the membrane’s surface, further developing a hydrophobic barrier below the membrane surface. Additionally, in human lenses with no detectable cataracts, α-crystallin was still able to bind to the lens membrane, indicating that α-crystallin binding itself may not be the sole cause of cataract development but may be required in lesser amounts for membrane stability and homeostasis.

Our study suggests that decreased Chol content in cataractous CM and NM results in increased binding of α-crystallin with increased CC and NC grade. Also, hydrophobicity of the male and female CM increases with an increase in CC grade, which suggests the decreased Chol content increases hydrophobicity on the membrane surface, resulting in increased α-crystallin binding with cataract development. Therefore, developing a strategy to increase Chol content in the lens membrane will likely reduce α-crystallin binding to the lens membrane and consequently prevent cataract development and progression. Furthermore, our data suggest that developing cholesterol derivative compounds that significantly decrease the hydrophobicity on the CM and NM surface would reduce α-crystallin binding to the membrane and likely prevent the development and progression of cataracts.

In this study, CM and NM isolated a single lens from the left and right eye of ten human lenses i.e., 64 yo male (one pair), 68 yo male (one pair), 68 yo female (one pair), 73 yo male (one pair), and 73 yo female (one pair) with varying CC and NC grade which were used for α-crystallin membrane binding studies where the sample size is relatively small, which is limitation of this study. The MSO and the physical properties (mobility, order, and hydrophobicity) of CM and NM with α-crystallin binding were investigated concerning the grade of CC and NC, in which each grade of CC and NC was associated with health history information such as diabetes, radiation treatment, smoking, and hypertension. However, to generalize the results and conclusions from this study, α-crystallin membrane binding studies from a large number of age-matched male and female human lenses (larger sample size) with varying CC and NC grades and associating each grade of CC and NC with health history information such as diabetes, radiation treatment, vitrectomy, corticosteroid therapy, and smoking would be needed, which our laboratory plans to pursue in the future.

The research reported in this paper shows the lipid compositional variances between the CM and NM and between differing grades of cataracts in single human lenses strongly modulates the binding of α-crystallin to the lens membrane. For future research, it would be beneficial to compare the membrane binding of α-crystallin with young non-cataractous lenses to better characterize how the lens lipid and Chol composition changes with aging and influences α-crystallin membrane binding and the physical properties of the membrane with such binding. Moreover, studying the interactions of α-crystallin in disease-matched lenses would be beneficial as it would help us to better understand how differing causes of cataracts affect the lens membrane. Of relevance in this study, we have used α-crystallin without any genetic mutation; however, earlier studies reported that genetic mutation of αA-crystallin (αAc) and αB-crystallin (αBc), i.e., αAc-R116C, αAc-R49C, αAc-G98R, αBc-R120G, and αBc-D140N cause cataracts [7,145,146,147,148,149,150,151,152]; however, the underlying mechanism of cataract formation is unknown. Previously, it has been reported that there was an increased binding of αAc-R116C with phospholipid membranes [153] and bovine CMs [8] compared to αAc; however, the MSO by αAc and αAc-R116C was not investigated. We plan to investigate the MSO by α-crystallin with genetic mutation of α-crystallin (i.e., αAc-R116C, αAc-R49C, αAc-G98R, αBc-R120G, and αBc-D140N) in our future research. Additionally, all of the donors in this study are Caucasian, but previous studies have shown different races may be at varying levels of risk for the development of cataracts [154,155]; therefore, studying the interactions of α-crystallin in lenses from individuals of different races may be beneficial to gain more insight into the impact race-related risk factors may have on the development of cataracts. Studies involving intrinsic membrane proteins show α-crystallin can bind to intrinsic proteins and affects the lipid interactions of α-crystallin [60,63,156], while other studies show that α-crystallin primarily binds to lipids [31,51,52,56,57,157]. Therefore, future studies are also needed to better understand the impact and synergistic effect of lens membrane lipids, Chol, CBDs, and intrinsic proteins on the interactions of α-crystallin with the lens membrane to better understand the development of human CC and NC.

## 4. Materials and Methods

### 4.1. Materials

Five pairs of human lenses from donors of different ages, i.e., 73-year-old (yo) (male, Caucasian), 73 yo (female, Caucasian), 68 yo (male, Caucasian), 68 yo (female, Caucasian), and 64 yo (male, Caucasian) were obtained from the Lions Eye Bank of Wisconsin and stored at −80 °C until total lipids (lipid plus Chol) isolation was performed. Lenses were removed in situ within an average of 16 h postmortem. A monophasic extraction protocol was used to extract the total lipids from the cortex and nucleus of each single lens, as further explained in Section 4.2 and previously [75,158]. Native bovine eye lens α-crystallin (C4163) and HEPES were obtained from Sigma Aldrich (St. Louis, MO, USA). 4-palmitamido-TEMPO (4PT) spin-label was acquired dissolved in chloroform from Avanti Polar Lipids, Inc. (Alabaster, AL, USA). Native bovine lens α-crystallin was dissolved in HEPES buffer (0.55 mM HEPES, pH = 7.4) and used without further purification.

### 4.2. Isolation of Cortical and Nuclear Lipids from Single Human Lens

The five pairs of human lenses (three male and two female) obtained from the Lions Eye Bank of Wisconsin were individually graded, using a binocular microscope, on a scale of 0–3 based on the opacity and yellowness for CC and NC, respectively, with a similar grading approach described by Zhao et al. [159]. The CC is based on the opacity of the lens with grades of 0, 1, 2, and 3 corresponding to no opacity, traces of opacity, dense opacity, and complete opacity, respectively. Similarly, NC are graded on the amount of yellowness in the lens, with grades of 0, 1, 2, and 3, corresponding to no detectable yellowness, mild yellowness, moderate yellowness, and severe yellowness, respectively. Two lab members performed grading independently to eliminate the bias in the grading approach. The CC and NC grades for the right eye lenses were as follows: 64 yo male (CC: 0, NC: 2), 68 yo male (CC: 3, NC: 2), 73 yo male (CC: 1, NC: 2), 68 yo female (CC: 3, NC: 2), and 73 yo female (CC: 1, NC: 3). Conversely, the CC and NC grade for each of the left lenses analyzed were: 64 yo male (CC: 0, NC: 2), 68 yo male (CC: 3, NC: 2), 73 yo male (CC: 2, NC: 3), 68 yo female (CC: 3, NC: 2), and 73 yo female (CC: 1, NC: 3). 

To begin isolating lipids from the cortex and nucleus of each lens, the lens was taken out of a −80 °C freezer and allowed to defrost at room temperature, followed by grading the lens for CC and NC as described above. Each lens was then decapsulated, and the cortex and nucleus were further separated based on differences in tissue consistency [75,160,161]. Once separated, cortical and nuclear lipids were isolated using methods based on a monophasic extraction protocol described previously [75,158]. In this process, ~2 mL of methanol is used to transfer the separated cortical and nuclear tissues into separate glass centrifuge tubes containing 10 mL of methanol. A Dounce homogenizer is then used to homogenize each tissue in the methanol, and methanol is then added to a final volume of 15 mL. Homogenized cortical and nuclear tissues were further sonicated three times for 15 s each, with a 30 s cooling period in ice between sonication cycles using probe-tip sonication (Fisher Scientific, Model 550, Waltham, WA, USA). The homogenized solutions were centrifuged (4 °C, 1 h, 5000 rpm) using an Avanti J26S XP centrifuge and JA-25.50 rotor (Beckman Coulter, Brea, CA, USA). The lipid-containing supernatant from each solution was then decanted into glass beakers, leaving a smaller layer remaining to avoid transferring pelleted impurities. The supernatant-containing beakers were placed on a hot plate (Fisher Scientific, Waltham, WA, USA) at 60 °C with a controlled stream of N2-gas to evaporate the methanol fully. Once dried, 10 mL of hexane and isopropanol (2:1 *v*/*v*) solutions were added to each beaker to dissolve the dried lipid films into solution. The lipid solutions were transferred into glass centrifuge tubes and sonicated with a probe-tip sonicator for 15 s to homogenize each solution further. The solutions were then recentrifuged (4 °C, 1 h, 5000 rpm), and the lipid-containing supernatant from each tube was again decanted into a fresh beaker. The decanted supernatants were evaporated at 60 °C using a controlled stream of N_2_-gas to a volume of 2 mL. The remaining solutions were transferred to fresh glass centrifuge tubes, and hexane and isopropanol (2:1 *v*/*v*) were added to each tube to a final volume of 5 mL. The tubes were centrifuged a final time (4 °C, 1 h, 5000 rpm) to remove any remaining impurities. The supernatant from each tube was transferred into weighted small glass tubes, and solutions were dried at 60 °C using a stream of N_2_-gas. Once dried, each tube was left in a vacuum overnight to remove any traces of hexane and isopropanol. The weight of each glass tube containing the dried lipid films was measured, and the total lipids (lipid plus Chol) from the cortex and nucleus of a single human lens was estimated. The total cortical and nuclear lipids isolated from a single human lens were estimated to be ~1.5 mg and ~1.2 mg, respectively. The cortical and nuclear lipids were dissolved in chloroform, maintaining 0.5 mg/mL of total lipids, and were stored at −20 °C.

### 4.3. Preparation of Small Unilamellar Vesicles (SUVs) from Cortical and Nuclear Lipids Isolated from a Single Human Lens

To prepare CM and NM, 600 µL of the total lipids (0.5 mg/mL) from a single human lens cortex or nucleus was mixed with 20 µL of a chloroform solution of 4PT spin-label (0.5 mM). 4PT spin-label concentrations were maintained at 1.37% (*w*/*w*) spin-label to total lipids mixture containing total cortical or nuclear lipids. Following mixing, N_2_-gas was used to dry the mixtures to a volume of ~75 μL. Once dried, ~360 μL of warm (~50 °C) buffer (0.55 mM HEPES, pH = 7.4) was added to the evaporated chloroform mixture. The rapid solvent exchange (RSE) method [162,163,164,165] was then used to prepare large multilamellar vesicles (LMVs) [165,166]. RSE is done by placing the buffer-chloroform mixture containing tube on a vortexer and connecting the tube to the sample manifold of the RSE equipment. The vortexer is then activated, the manifold valve opened to a vacuum system set at ~25 Torr, and ran for ~2 min. After 2 min, the sample is flushed with argon and withdrawn from the RSE equipment. The produced LMVs are then developed into small unilamellar vesicles (SUVs) using probe tip sonication (Fisher Scientific, New Plainville, MA, USA, Model 550) for 25 cycles of 10 s sonication followed by 50 s of cooling on ice and 30 s heating in ~50–60 °C water bath. The hydrodynamic radius of the SUVs for each sample was determined using dynamic light scattering (DLS) and further used in Section 4.4 to calculate the MSO by α-crystallin. A total of 125 µL of CM or NM containing 0.15 mg of total lipids was mixed with 19.1 µL (3.54 mg/mL) of α-crystallin in HEPES buffer (0.5 mM HEPES, pH = 7.4), maintaining 23.06 μM α-crystallin concentration in incubation volume of 144.1 µL. For the control samples without α-crystallin, 19.1 µL of HEPES buffer was added to 125 µL of CM or NM containing 0.15 mg of total lipids so that the incubation volume remains the same as that of CM and NM containing α-crystallin. Our prior studies on α-crystallin binding with model eye lens lipid membranes and isolated bovine lenses showed α-crystallin binding to saturate at ~23.06 μM, which is where the initial value was derived [70,75]. Additionally, to verify the saturation of α-crystallin binding with isolated human lens membranes, multiple experiments were conducted with male human NM at increasing α-crystallin concentrations, and α-crystallin association additionally appeared saturated at ~23.036 μM. The mixtures (α-crystallin and CM or NM) were incubated at 37 ºC with gentle shaking (150 rpm) for 16 h in a benchtop incubator (Corning Inc., Corning, NY, USA) to allow for α-crystallin binding saturation [66,67,68,70,75]. After incubation, samples were concentrated by evaporating the solvent with the RSE device and were used for EPR measurements. 

### 4.4. The Electron Paramagnetic Resonance (EPR) Spin-Labeling Method to Investigate the α-Crystallin Binding to Human Cortical and Nuclear Membranes Isolated from a Single Lens

The incubated samples were loaded into a gas-permeable methylpentene polymer (TPX) capillary for continuous wave (CW) electron paramagnetic resonance (EPR) using an X-band Bruker ELEXSYS 500 spectrometer (Bruker Corporation, Billerica, MA, USA). Measurements were taken at either 37 °C to obtain information on α-crystallin binding with the CM and NM and the consequential effects of binding on the membrane’s mobility and order near the headgroup regions with α-crystallin binding, or at −165 °C to obtain the hydrophobicity near the membrane head group region with α-crystallin binding. All measurements were taken using a 1.0 mm internal diameter (i.d.) TPX capillary and a constant stream of N_2_-gas was used to deoxygenate the samples and maintain the temperature. However, experiments at −165 °C also required liquid N_2_ to maintain the low temperature. The EPR spectra from the measurements taken at 37 °C were done with a modulation amplitude of 1.0 G and an incident microwave power of 8.0 mW. Measurements taken at −165 °C were done with a modulation amplitude of 3.0 G and incident microwave power of 2.0 mW. The EPR spectra in the presence and absence of α-crystallin were normalized to the peak-to-peak intensity of each spectrum’s central field line to begin analyzing the interactions of α-crystallin with the CM and NM.

**Figure 9:**
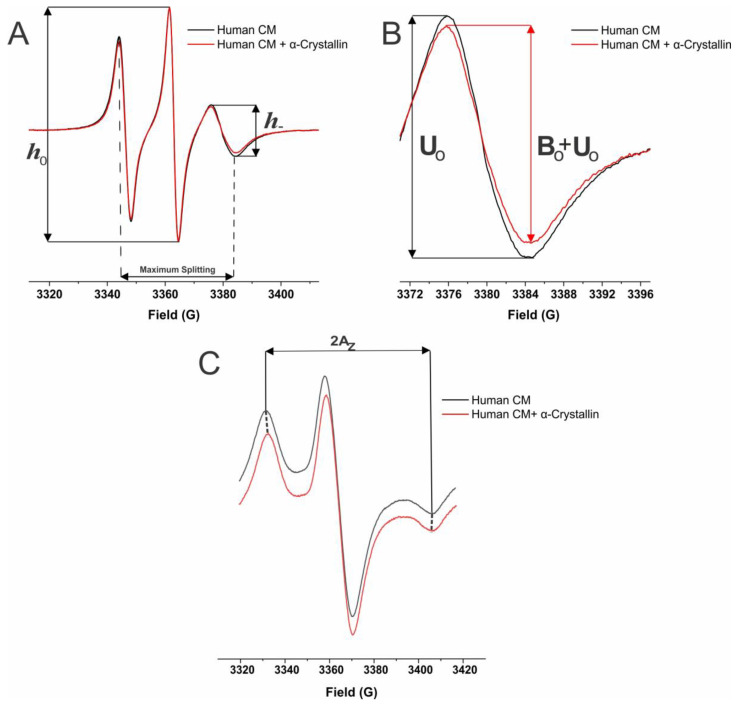
(**A**) EPR spectra, taken at 37 °C, of 4PT in 64 yo male right eye lens CMs in the absence of α-crystallin (black) and with 23.036 μM α-crystallin (red). The amount of cortical total lipids (lipid plus Chol) used in CM sample was 0.15 mg. The ratio of the peak-to-peak intensity of the high-field line (h_−_) and the central line (h_0_) provides the mobility parameter (h_−_/h_0_). The horizontal distance between the low- and high-field lines provides the maximum splitting. (**B**) Magnified image of the high-field line of the EPR spectra shown in (**A**), representing the unbound (U_0_) and unbound plus bound (U_0_ + B_0_) contributions. The change in the peak-to-peak intensity of the high-field line of the EPR spectra was used to calculate the percentage of the membrane surface occupied (MSO) by α-crystallin. (**C**) EPR spectra, taken at −165 °C, of 4PT in a 73 yo male left eye lens CM in the absence of α-crystallin (black) and with 23.036 μM α-crystallin (red), showing the horizontal distance between the low-field and high-field lines, being used to measure 2A_Z_, which is a measure for hydrophobicity.

Displayed in Figure 9A is a demonstrative EPR spectra, taken at 37 °C for the 4PT spin-label in the 64 yo male right eye lens CM both in the absence (black) and presence of α-crystallin (red). From these spectra, we can obtain information on the MSO, mobility parameter, and maximum splitting. As further depicted by the dotted lines, the distance in peaks from the low-field to the high-field lines gives us the values for the maximum splitting. Maximum splitting values provide information about the order on the membrane surface with α-crystallin binding [75]. Further shown by the solid lines, the ratio of the peak-to-peak height of the high-field line (h_−_) and the central line (h_0_) provides us with the mobility parameter, which provides information about the mobility on the membrane surface with α-crystallin binding [75]. Shown in Figure 9B is the zoomed-in high-field EPR lines of the spectra displayed in Figure 9A, and the red line in Figure 9B shows that α-crystallin binding to the CM decreased the peak-to-peak intensity of the high-field EPR line relative to the control membrane (black line). Previously, we have observed a similar decrease in the peak-to-peak intensity of a in high-field EPR line with α-crystallin binding to bovine CM and NMs [75]. 

As further depicted in Figure 9B, the high-field line of the EPR spectra from the control membranes (CM without α-crystallin) was used as an unbound contribution (U_0_), and the high-field line of the EPR spectra from CM with α-crystallin binding was used as an unbound plus bound (U_0_ + B_0_) contribution. This information was then used to calculate the percentage of 4PT spin-labels at the surface of the membrane affected by α-crystallin binding as previously described [66,67,68,75]:

(1) $$\%\;4PT\;\mathrm{spin-labels}\;affected=\left\{\frac{U_{0}-\left(U_{0}+B_{0}\right)}{U_{0}}\right\}\times 100\%$$

DLS measurements taken on a DynaPro instrument (Wyatt Technology Corp., Santa Barbara, CA, USA) using regularization methods (Dynamics software, version 7) were used to determine the hydrodynamic radius of the SUVs for all individual CM and NM samples. Individual radius data were used to calculate the MSO by α-crystallin for each sample. DLS measurements showed the average radius of the CM vesicles to be ~59 nm and the NM vesicles to be ~58 nm. Based on these radii, ~54% of the 4PT molecules were on the outer surface of the CM and NM. As the only 4PT spin-labels affected by the binding of α-crystallin were those on the outer surface, the corrected percentage of 4PT spin-labels affected by α-crystallin or MSO by α-crystallin was estimated by multiplying Equation (1) by the corrections factor. A correction factor of 100/54 was used for the CM and NM samples to evaluate the corrected % 4PT spin-label affected by α-crystallin, giving us the MSO of α-crystallin [66,67,68,70,75]. An example calculation for the CM with the correction factor reads as follows:

(2) $$\%\;Membrane\;surface\;occupied\;(MSO)=(\%\;4PT\;\mathrm{spin-labels}\;affected)\times \left(\frac{100}{54}\right)$$

The z-component of the hyperfine interaction tensor (A_z_) for the 4PT spin labels in the CM and NM was measured from samples frozen at approximately −165 °C with the use of liquid nitrogen. Displayed in Figure 9C is an EPR spectra of the 73 yo male left eye lens CM membranes taken at approximately −165 °C with and without α-crystallin, which shows the horizontal distance between the low-field line and the high-field line. This horizontal measurement provides the 2A_z_ value, which is a measure of hydrophobicity [38,70,75,167,168,169]. The higher the 2A_z_ value from the 4PT spin-label in a membrane, the lower the hydrophobicity on the surface of the membrane [75].

### 4.5. Statistics

For statistical significance all experiments were repeated at least three times, and at least three different preparations of the samples were used for repetitive experiments. Furthermore, all results are presented as the mean ± standard deviation (σ). Statistical significance of MSO, mobility parameter, maximum splitting, and hydrophobicity values were evaluated using the student’s *t*-test, with a *p* value ≤ 0.05 considered statistically significant unless otherwise stated.

## Figures and Tables

**Table 1:** Increase in MSO by α-crystallin with the development of cataracts.

Male- Right Eye Lens		Male- Left Eye Lens	
Increase in cataract grade	Increase in MSO	Increase in cataract grade	Increase in MSO
CC (0) ^a^ to CC (1)	3.62	CC (0) to CC (2)	3.73
CC (0) to CC (3)	6.41	CC (0) to CC (3)	10.13
CC (1) to CC (3)	2.79	CC (2) to CC (3)	6.40
NC (2) to NC (2)	0.02	NC (2) to NC (3)	6.35
Female- Right Eye Lens		Female- Left Eye Lens	
Increase in cataract grade	Increase in MSO	Increase in cataract grade	Increase in MSO
CC (1) to CC (3)	15.53	CC (1) to CC (3)	9.64
NC (2) to NC (3)	3.74	NC (2) to NC (3)	3.59

^a^ The number in parenthesis indicates the grade of the cataract.

**Table 2:** Change in mobility parameter (MP) with the development of cataracts.

Male- Right Eye Lens		Male- Left Eye Lens	
Increase in cataract grade	Change in MP	Increase in cataract grade	Change in MP
CC (0) ^a^ to CC (1)	0.011 (decrease)	CC (0) to CC (2)	0.016 (decrease)
CC (0) to CC (3)	0.018 (decrease)	CC (0) to CC (3)	0.023 (decrease)
CC (1) to CC (2)	0.007 (decrease)	CC (2) to CC (3)	0.007 (decrease)
NC (2) to NC (2)	0.009 (decrease)	NC (2) to NC (3)	0.001 (increase)
Female- Right Eye Lens		Female- Left Eye Lens	
Increase in cataract grade	Change in MP	Increase in cataract grade	Change in MP
CC (1) to CC (3)	0.021 (decrease)	CC (1) to CC (3)	0.014 (decrease)
NC (2) to NC (3)	0.028 (increase)	NC (2) to NC (3)	0.02 (increase)

^a^ The number in parenthesis indicates the grade of the cataract.

## Data Availability

Data are contained within the article.

## Abbreviations

EPR	electron paramagnetic resonance
CM	cortical membrane
NM	nuclear membrane
CC	cortical cataract
NC	nuclear cataract
MSO	percentage of membrane surface occupied
4PT	4-palmitamido-TEMPO
PL	phospholipid
SL	sphingolipid
Chol	cholesterol
CBD	cholesterol bilayer domain
